# A rare case of severely elevated septic parameters caused by intercurrent juvenile rheumatoid arthritis despite dual trauma surgery

**DOI:** 10.1093/jscr/rjad168

**Published:** 2023-04-12

**Authors:** Lennard M Wurm, Julian R Andresen, Justyna Reinke, Denis Poddubnyy, Wolfgang Ertel, Michal W Jagielski

**Affiliations:** Department of Traumatology and Reconstructive Surgery, Charité – Universitätsmedizin Berlin, corporate member of Freie Universität Berlin, Humboldt-Universität zu Berlin and Berlin Institute of Health, Berlin, Germany; Medical Faculty Heinrich-Heine University and University Hospital Düsseldorf, Düsseldorf, Germany; Department of Traumatology and Reconstructive Surgery, Charité – Universitätsmedizin Berlin, corporate member of Freie Universität Berlin, Humboldt-Universität zu Berlin and Berlin Institute of Health, Berlin, Germany; Department of Traumatology and Reconstructive Surgery, Charité – Universitätsmedizin Berlin, corporate member of Freie Universität Berlin, Humboldt-Universität zu Berlin and Berlin Institute of Health, Berlin, Germany; Department of Gastroenterology, Infectious Diseases and Rheumatology, Charité – Universitätsmedizin Berlin, corporate member of Freie Universität Berlin, Humboldt-Universität zu Berlin and Berlin Institute of Health, Berlin, Germany; Department of Traumatology and Reconstructive Surgery, Charité – Universitätsmedizin Berlin, corporate member of Freie Universität Berlin, Humboldt-Universität zu Berlin and Berlin Institute of Health, Berlin, Germany; Department of Traumatology and Reconstructive Surgery, Charité – Universitätsmedizin Berlin, corporate member of Freie Universität Berlin, Humboldt-Universität zu Berlin and Berlin Institute of Health, Berlin, Germany

**Keywords:** infection parameters, juvenile idiopathic arthritis, trauma surgery, procalcitonin

## Abstract

Procalcitonin (PCT) and C-reactive protein (CRP) are considered markers used in clinical practice to differentiate bacterial infections from autoimmune origin. Here we evaluate a rare case of a male patient diagnosed with juvenile idiopathic arthritis. The patient presented repeatedly to our department with atraumatic femoral head necrosis, traumatic medial femoral neck fracture and peri-implant femoral fracture. While undergoing repeated surgical interventions including a removal of osteosynthesis material and total endoprosthesis of his right hip including double subtrochanteric osteotomy, the patient developed drastically increasing infection parameters of PCT and CRP. After a completely inconspicuous revision we revealed the untypical genesis of a rheumatic cause. Consequently, we emphasize this etiology to be considered in further decision making for trauma surgery.

## INTRODUCTION

Surgical and orthopedic care in rheumatic patients necessitates various differential diagnoses regarding infectious parameters. The distinction between autoinflammatory and bacterial genesis determines different treatment regimens making their knowledge essential. A variety of infectious parameters are used to guide decision making. These include Procalcitonin (PCT) and C-reactive protein (CRP). PCT is physiologically produced by neuroendocrine cells such as stomatic C cells of the thyroid gland, pulmonary tissue and pancreas as a precursor to calcitonin. PCT values elevated above 0.5 μg/l imply serious infection by a hitherto unknown mechanism [1]. A high sensitivity of 65% and specificity 91% for bacterial infections make it a surrogate marker for septic infections [2]. Only few cases describe elevated PCT values with rheumatic cause [3] due to false positive semiquantitative immunochromatography assays. CRP as an acute-phase protein serves as a stress and infection parameter. CRP may be elevated in rheumatologic diseases, but severe increases are usually indicative for bacterial genesis [4]. The general evidence indicates use of both parameters as diagnostic differentiators for septicemia etiology [5].

Based on the importance of correct treatment, we discuss a rare case where infectious parameters may lead to a wrong diagnosis. This case is intended to demonstrate the impact of rheumatologic disease in the trauma surgery setting.

## CASE REPORT

Here we evaluate a rare case of a 34-year-old Ukrainian patient after a complex disease progression and medical history. The patient with constitutionally short stature of 156 cm and 46 kg suffers from HLA-B 27 positive seronegative polyarticular juvenile idiopathic arthritis (JIA) since his 4th year of birth. In the course of his morbidity, he developed significant dystrophies and deformities of the proximal extremities, as well as postural pelvic and shoulder obliquity. He is diagnosed with congenital varus deformity of his right femur with hip socket dysplasia of Graf grade 2, the right knee joint was fused during an attempt of leg lengthening in the 12th year of birth leading to a length difference of 13 cm between both legs. Incidentally, the patient suffers from a variety of comorbidities as, most relevantly, multidrug-resistant tuberculosis initially diagnosed 6 years ago and alimentary marasmus, normocytic normochromic anemia and chronic renal insufficiency eluding the vulnerability for infections.

The patient is under treatment due to exacerbations of his JIA, his rheumatologic disease was managed by Etanercept, Prednisolone and Sulfasalazine and switched to Tofacitinib 2 years ago with constant CRP values of 60 mg/l.

**Figure 1:**
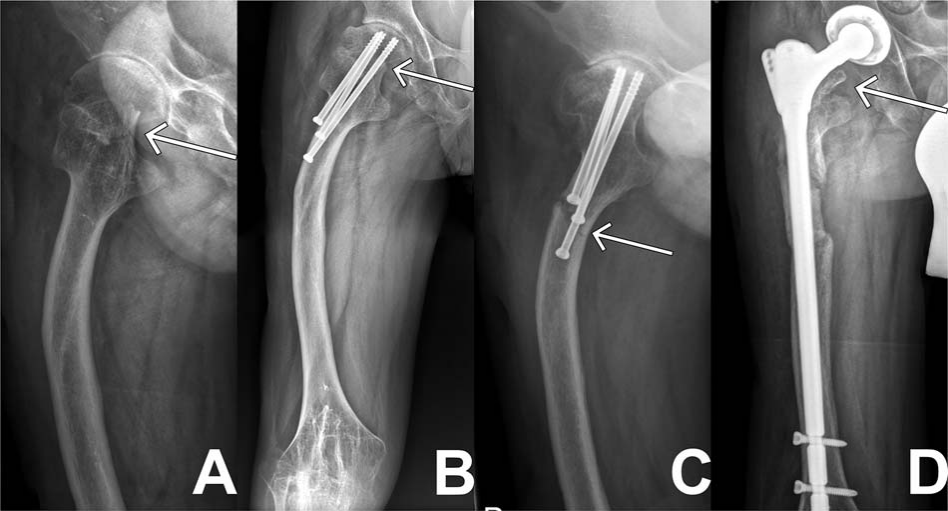
X-ray sequences of the right hip in different resolutions. (**A**) Representation of the medial femoral neck fracture before the first intervention as highlighted by arrow. (**B**) Osteosynthesis intervention of hip is highlighted by arrow few months after intervention. In addition, femoral deformity and knee rigidity are recognizable. (**C**) Periosteosynthetic fracture of the femoral shaft shortly before intervention. (**D**) Follow-up image of the inserted prosthesis material shows regular position.

Initially, the patient presented to our joint consultation with a femur head necrosis ARCO 4, a femur deformity and arthritic hip changes, thus we initialized an elective total endoprosthesis (TEP) of the right hip. Before the operation, the patient was represented to our emergency department after a falling on the right hip with inner rotation pain. A medial femoral neck fracture of Garden 4 as seen in Fig. 1(A) was diagnosed. Because of his young age and the presented situation, we decided to perform a joint-presenting closed reduction and internal triple screw fixation of the proximal femur using 3 × 6.5 mm titanium cannulated screws the following day.

The following week, the patient presented to the emergency department again with severe progressive wrist and ankle pain. Having diffuse myalgias and arthralgias with CRP values of 200 mg/l, we assumed the onset of JIA due to perioperative pausing of the Tofacitinib medication. His conditions improved after corticosteroid boost therapy with a dosage of 100 mg per day.

**Figure 2:**
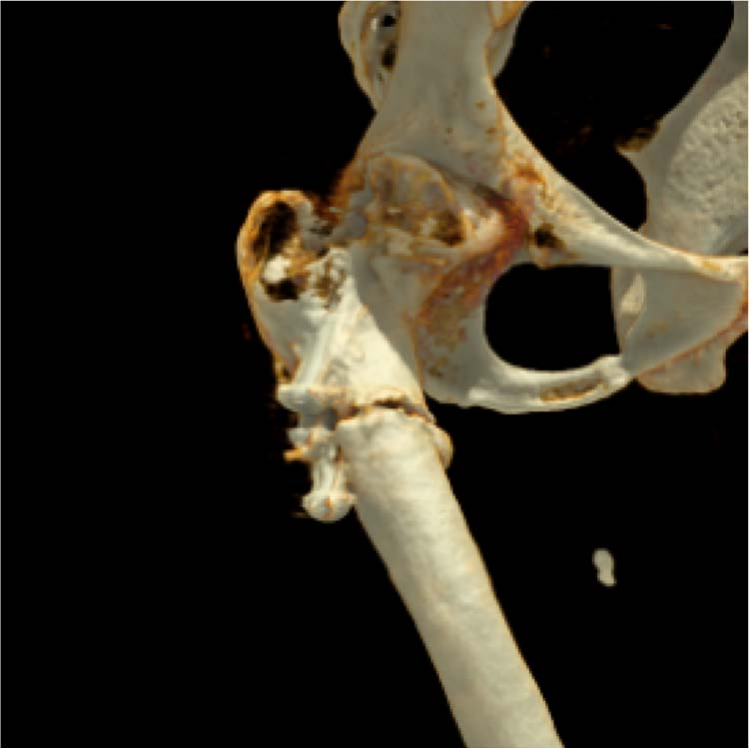
Three-dimensional reconstruction of computer tomography shows the patient’s periosteosynthetic fracture.

Two months later, the patient was represented to the emergency department after stumbling with progressive pain being conservatively treated due to bland diagnostics. Despite the good progress as seen in follow ups exampled in Fig. 1(B), the patient presented after another two months with atraumatic intense immobilizing pain of the right hip. Radiological diagnosis revealed a periosteosynthetic femoral shaft fracture as seen in Figs 1(C) and 2. The indication for the need of a TEP was given.

Hereby, the inserted material was completely removed, a double sub trochanteric repositioning osteotomy of the right femur, open surgical fracture reduction by means of cerclage (Arthrex, 2× Fibertapecerclage), an autologous and allogeneic spongiosaplasty of femoral shaft and an implantation of a modulatory cementless TEP using the smallest specifications was operated. Intraoperative specimen revealed existence of *Staphylococcus Aureus* on all three explanted screws. We further initiated an antibiosis with Meropenem, Rifampicin and Spaphylex in accordance with resistance protocol perioperatively.

In the following days the patient’s pain and infection parameters increased. With the suspicion of infection, open joint lavage, debridement with drain and Vancomycin insertion was performed 2 weeks later. The intraoperative specimen didn’t reveal any microbiological findings. The clinical situation and infectious parameters developed worse. The CRP values progressively increased reaching its peak shortly after revision at 454 mg/l, the PCT values reached levels of 1.94 μg/l when measured over several days. After revision, appropriate antibiotic therapy and proper prosthesis inlay in radiological controls, an exacerbation of the JIA was now suspected because of hints like diffuse myalgia and arthralgia with further increasing CRP and PCT parameters. A prednisolone shock therapy was initiated. Under this therapy, pain and infection parameters decreased, thus confirming the assumption. The patient could be discharged to outpatient follow-up treatment. The following controls confirmed a good clinical course as seen in Fig. 1(D).

## DISCUSSION

As described in this case, the infection parameters must be considered in a differentiated manner. We discuss a patient with a complex trauma surgical history and relevant risk factors for a septic infection. The most probable cause of increasing pain and rising infection parameters as well as the detection of *S. Aureus* suggested the rising of a septic genesis. Revision with antibiotic therapy and negative specimen rejected this cause. The fact that this genesis can be traced back to the JIA highlights the inclusion of intercurrent causes leading to levitating PCT and CRP parameters. As we introduced, false-positive laboratory methods have been described in rare cases [3]. However, in our case we find this unlikely to affect all parameters on repeated days. Despite the evidence which emphasizes these parameters as differential markers for septic genesis, this case should serve as reference in future decision making.

## AUTHORS’ CONTRIBUTIONS

All authors have read and agreed to the published version of the manuscript.

## CONFLICT OF INTEREST STATEMENT

The authors declare no competing interests.

## FUNDING

This research did not receive any specific grant from funding agencies in the public, commercial or not-for-profit sectors.

## DATA AVAILABILITY

Data generated during and/or analyzed during the current study are available from the corresponding author on reasonable request.
